# The effects of prosthetic foot type and visual alteration on postural steadiness in below-knee amputees

**DOI:** 10.1186/1475-925X-13-23

**Published:** 2014-03-05

**Authors:** Nooranida Arifin, Noor Azuan Abu Osman, Sadeeq Ali, Wan Abu Bakar Wan Abas

**Affiliations:** 1 Department of Biomedical Engineering, Faculty of Engineering, University of Malaya, 50603 Kuala Lumpur, Malaysia

**Keywords:** Postural steadiness, Computerized posturography, Prosthetic foot, Stability indexes, Transtibial amputees, Unperturbed standing, Visual input

## Abstract

**Background:**

Achieving independent upright posture has known to be one of the main goals in rehabilitation following lower limb amputation. The purpose of this study was to compare postural steadiness of below knee amputees with visual alterations while wearing three different prosthetic feet.

**Methods:**

Ten male below-knee amputees were instructed to stand quietly on the Biodex® balance platform while wearing solid ankle cushion heel (SACH), single axis (SA) and energy storage and release (ESAR) prosthetic foot under different visual input conditions (eyes-opened and eyes-closed). The overall stability index (OSI), anterior- posterior stability index (APSI), and medial-lateral stability index (MLSI) were computed. Perceived balance assessment of each foot was evaluated using Activities-specific Balance Confidence (ABC) score.

**Results:**

The findings highlights that SACH showed lowest overall stability index (indicating less body sway) during eyes-opened (OSI: SACH = 1.09, SA = 1.58, ESAR = 1.59) and SA showed lowest overall stability index during eyes-closed (OSI: SACH = 2.52, SA = 2.30, ESAR = 2.76) condition. However, overall stability indexes between foot types did not differ significantly during eyes-opened or eyes-closed (p = 0.651). There was a trend of instability which occurred more in medial-lateral compared to anterior-posterior direction for all foot types, with significant result in ESAR foot(eyes-opened: MLSI = 1.59, APSI = 0.65, p = 0.034; eyes-closed: MLSI = 2.76, APSI = 1.80, p = 0.017, respectively). When comparing between visual conditions, stability score was significantly higher during eyes-closed compared to eyes-opened situations for SACH and ESAR foot (eyes-closed vs opened; SACH OSI: 3.43 vs 1.71, p = 0.018 and MLSI: 3.43 vs 1.71, p = 0.018; ESAR OSI: 3.58 vs 1.86, p = 0.043 and APSI: 1.80 vs 0.65, p = 0.027).

**Conclusions:**

The results of this study suggested postural steadiness in below-knee amputees was not affected by the types of prosthetic foot during quiet upright standing, but was significantly affected when visual cues was absent.

## Background

The ability to maintain postural stability is the foundation of achieving independent standing and walking [1]. It is a complex task that integrates somatosensory (proprioceptive, cutaneous and joint), visual and vestibular inputs along with motor coordination to maintain the center of mass (CoM) within the base of support [2-4]. Any deficits in these components will result in poor control of body posture, which is often associated with the risk of falling and has been identified as a major health problem [5]. In people with lower limb amputations, they must compensate for the challenging task in maintaining postural stability by increasing dependence on visual and vestibular information [3]. Due to the important role of visual information, postural stability assessment with eyes-closed condition is necessary to determining the utilization of other sources of sensory information during postural control in addition to the eyes-open condition which serves as baseline clinical assessment [6]. In fact, previous study showed that the absence of visual input will increase the postural sway and asymmetry of stance in below-knee amputees [7].

In able-bodied person, the motor coordination responsible for postural stability maintenance consists of ankle and hip strategies which produce corrective torque in order to counter the destabilizing torque due to gravity that causes deviation of the CoM [8]. In the absence of perturbation, the muscle contracts eccentrically to resist the gravitational forces. However, in order to maintain postural stability during perturbation, concentric muscle contraction is essential. Hence, stiffer muscles potentially increase the efficiency of postural control mechanism. Researchers theorized that stiffness of the ankle muscle might play an important role in maintaining balance and joint stability [2,9]. However, loss of muscular structures as results of below-knee amputation causes deficits in sensory input from proprioceptive component at the feet and ankle. As a result, amputees exhibit a higher incidence of falling than able-bodied people because of the former’s deficits in controlling horizontal movements in medial-lateral or anterior-posterior directions [10].

Consequently, to substitute for the loss of the ankle-foot complex, the prosthetic foot is prescribed for the amputees. Along with advancements in technology, prosthetic foot has gone through tremendous transformations in terms of design and materials used. From previous postural balance assessment in the amputee subjects, researchers suggested that reduced sway may be due to the relatively stiff ankle of the prosthetic foot which limits the dorsiflexion or plantarflexion movement [11,12]. However, the effect of such stiffness to the postural balance remains unclear due to the variations in types of prosthetic feet tested in such studies that may have had influenced their balance performance.

Although balance confidence and stability has shown to associate with walking performance and social activity [10], studies on postural balance with different foot category are scarce compared with research on other biomechanical areas [13]. The primary purpose of this study is to systematically assess the influence of three different prosthetic foot types to the overall, medial-lateral, and anterior-posterior control of postural steadiness in person with below-knee amputation. The secondary purpose was to compare postural steadiness during quiet standing when visual inputs were altered.

## Methods

### Participants

A convenience sample of ten male unilateral below-knee amputees gave written consent to participate in this study. All subjects had at least one year experience in current prosthesis and able to walk without the use of assistive device. Subjects with visual or vestibular impairment, residuum pain, other neurological deficits or musculoskeletal injury were excluded. This study was approved by the Institutional Review Board in accordance with the Helsinki Declaration. Subjects are all recruited via the University of Malaya Medical Centre that undergone the same rehabilitation programs. In this study, each subject served as his own control. The participants’ demographic information is shown in Table 1.

**Table 1:** Participant characteristics

**Subject**	**Age (y)**	**Height (m)**	**Mass (kg)**	**Cause of amputation**	**Mobility grade**^**†**^	**Time since amputation (y)**	**Prosthetic foot**	**Suspension**	**BBS (total 56)**
1	59	1.71	75	Diabetic	K2	6	SA	PTB with pelite	52
2	23	1.62	88	Trauma	K3	2	SA	PTB with pelite	56
3	45	1.78	84	Trauma	K3	25	SA	PTB with pelite	56
4	52	1.67	64	Trauma	K3	5	ESAR	TSB with pin lock	56
5	42	1.72	58	Trauma	K3	9	SA	TSB with pin lock	56
6	38	1.75	100	Diabetic	K2	5	SA	TSB with pin lock	56
7	44	1.77	109	Diabetic	K2	3	ESAR	TSB with pin lock	49
8	25	1.65	55	Tumor	K3	3	ESAR	TSB with pin lock	56
9	61	1.62	69	Diabetic	K2	7	SA	TSB with pin lock	51
10	59	1.66	68	Diabetic	K2	6	SA	TSB with pin lock	41
Mean	44.8	1.70	77.0			7.1			52.9
SD	13.5	0.06	17.9			6.6			4.9
*p*-value	0.325	0.391	0.504						

^†^Based on Medicare K-level.

PTB: Patellar tendon bearing socket, TSB: Total surface bearing socket, BBS: Berg Balance Score. Statistically significant of differences between the study groups was set as p < 0.05.

### Foot selection

**Figure 1:**
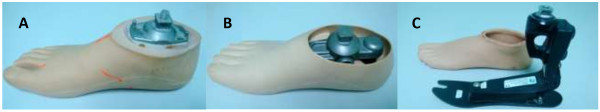
Types of prosthetic foot used on this study: (A) Solid ankle cushion heel (SACH) foot, (B) Single axis (SA) foot and (C) Energy storage and release (ESAR) foot.

Three different foot types were tested: solid ankle cushion heel foot (Enjoylife, Fujian, China), single-axis foot (Enjoylife, Fujian, China) and energy saving and return foot Talux® (Ossur, Reykjavik, Iceland), as shown in Figure 1. The solid ankle cushion heel foot (SACH) is a non-articulating foot that has wooden keel and high-density rubberized foam cushioned heel. The single-axis foot consists of a single hinge that permits 15-20° of plantarflexion and 5-8° of dorsiflexion motion. The energy saving and return foot Talux® is a Flex-Foot with J-shaped multiaxial ankle and heel-to-toe carbon fibre footplate designs. Each test foot was attached to the patient’s existing prosthesis and optimally aligned by the same registered prosthetist. After completed the static and dynamic alignments, subjects walked for 15 minutes to familiarize with the foot. Each tested foot was prescribed according to the subject’s foot size and body weight, with addition of activity level for the Talux® foot. Subjects were tested with their own socket and suspension components throughout the study. Although the structure of the prosthetic foot can be seen by the subject, the mechanical differences between the test feet were not revealed. Each subject wore their own covered shoe and the same shoe was used for each prosthetic feet. The heel height ranged from 2–2.5 cm between subjects.

### Procedure

Familiarization of the test procedures was conducted during the first visit. The prosthetist evaluated and ensured that the subjects’ existing prosthetic sockets and components were well fit before the testing trials. Subjects completed Short Form Health Survey (SF12v2) to evaluate the quality of life status of the subjects [14] to confirm that their postural stability is not affected by confounding factors from poor mental and physical conditions. To ensure similar balance status of all subjects, the Berg balance test [15] was conducted to assessed functional balance performance. A score of 0–20 indicates a high risk, 21–40 indicates a medium risk, and 41–56 indicates a low risk of falling. Subjects who failed to maintain equilibrium during the test were excluded from the study.

The first foot type was fitted during the first visit. After one week of accommodation period, subjects return to the laboratory for assessment. This period was reported sufficient for below-knee amputees before functional assessment of the prosthesis [16]. All subjects completed the Activities-specific Balance Confidence (ABC) scale [10] at each testing session to rate their balance confidence of a particular test foot. The overall score out of 100 was calculated by taking the average score of all items. After all test procedures were completed for the first foot, the foot was removed and replaced with the second test foot. Subsequently, the third test foot was attached to the prosthesis in the following week. The test was counterbalance across subjects to negate order effects. Finally, subjects attended the final visits to change the test foot with their original foot.

### Balance testing

Postural stability indexes during quiet standing with eyes-opened (EO) and eyes-closed (EC) were measured using the Biodex Stability System (BSS) (Biodex Medical System, Shirley, NY, USA) for its known reliability in objective assessment of postural stability [17,18]. This device consists of a circular platform with series of strain gauges which can be used to assess subject’s control of balance on either static or unstable surface condition [19]. From the CoM excursion about the anterior- posterior and medial- lateral axes from the center point, the BSS measures the overall stability index (OSI), anterior-posterior stability index (APSI) and medial- lateral stability index (MLSI). Furthermore, OSI was suggested as the best balance indicator [20]. The platform was integrated with computer software (Biodex, Version 3.1, Biodex Medical Systems) that enables the device to calculate the stability indexes. Since BSS assessed deviations from center, lower index score indicated greater stability. Subjects were instructed to step on the BSS platform and stood with a standardized position with each foot positioned 17 cm between the heel centres and 14˚ between the long axes of the feet to eliminate between-subject variability or biased results during balance testing [21]. To ensure this standardized position was maintained accurately for each test across all subjects, the positions were marked on the balance platform. During the test, subjects were asked to maintain their arms alongside the body, and look straight ahead at a point on the wall approximately 1.5 m away at eye level to prevent vestibular distraction and head movement. The platform was then locked into stable position, and foot placement was recorded as manufacturer’s guidelines [19]. Each testing trial lasted for 20 seconds and three testing trials were measured for reliable measures [17], both with eyes open and eyes closed. A standardized instruction was given to all subjects to “stand as still as possible” to ensure high consistency in their body sway during static posturography assessment [22]. Subject was allowed to a 30 seconds rest periods in a sitting position between the trials and were instructed not to change the position of their feet on the platform. The handrails at both sides of the BSS were positioned and could only be used to prevent falling if the subjects totally lost their balance. In addition, an assistant stood at the back of the subject for additional safety. In the event of malposition of the feet or loss of balance, the trial was deleted and data collection was continued until all trials were completed.

### Statistical analysis

All data were initially screen for normality of distribution by using the Shapiro Wilk’s test. Therefore, non-parametric statistical analyses were adopted. The Friedman’s repeated measures test were used to compare the overall ABC score and stability indexes for the three prosthetic feet. When differences were identified between groups, post-hoc pairwise comparison was conducted to determine where the significant differences occurred. The Wilcoxon-signed rank test was used to compare between EO versus EC conditions and APSI versus MLSI score for each prosthetic foot. Statistical analysis was performed using SPSS v16.0 (SPSS Inc., Chicago, IL, USA), with level of significance was set at p < 0.05 for all analysis.

## Results

### Participants’ characteristics

The mean age, weight and height for all ten participants were shown in Table 1. No significant differences were observed among the amputees in terms of age, height, and body mass. The Berg balance score indicated that all participants have a low risk of falling. According to the Medicare Functional Classification Level [23], participants engaged in K2-K3 activity level.

### Comparison between prosthetic foot types

The average and mean values for all outcome parameters with the significant differences observed are depicted in Table 2. When Friedman test were made between prosthetic foot types (SACH vs SA vs ESAR), the stability indexes score (OSI, APSI, MLSI) revealed non-statistically significant differences during both eyes-opened condition (p = 0.651, p = 0.607, p = 0.317 respectively) and eyes-closed condition (p = 0.651, p = 0.630, p = 0.891 respectively). The MLSI was statistically higher than APSI for ESAR foot in both eyes-opened and eyes-closed conditions (p = 0.034 and p = 0.017, respectively).

**Table 2:** The mean and (standard deviation) of stability indexes score and ABS score for three types of prosthetic foot during eyes-opened and eyes-closed conditions

**Outcomes parameters**	**Visual cues**	**Types of prosthetic foot**		
		**SACH**	**SA**	**ESAR**
APSI mean (sd)	EO	1.08 (1.02)*	0.80 (0.68)	0.65 (0.34)^¥^*
	EC	1.89 (0.96)	1.33 (0.61)	1.80 (1.03)^¥^
MLSI mean (sd)	EO	1.09 (0.92)*	1.58 (1.94)	1.59 (1.35)^¥^*
	EC	2.52 (1.19)	2.30 (1.18)	2.76 (1.37)^¥^
OSI mean (sd)	EO	1.71 (1.25)*	1.90 (1.99)	1.86 (1.34)*
	EC	3.43 (1.17)	2.91 (1.06)	3.58 (1.49)
ABC score mean (sd)		79 (13.8)^a^	86.1 (7.5)^b^	90.6 (7.1)^a,b^

^¥^p < 0.05: significant difference in comparison to MLSI and APSI.

*p < 0.05: significant difference in comparison to eyes-opened (EO) and eyes-closed (EC).

^a^p < 0.05: significant difference when compared with ABC score between SACH and ESAR using post-hoc analysis.

^b^p < 0.05: significant difference when compared with ABC score between SA and ESAR using post-hoc analysis.

### Comparison between eyes-opened and eyes-closed

**Figure 2:**
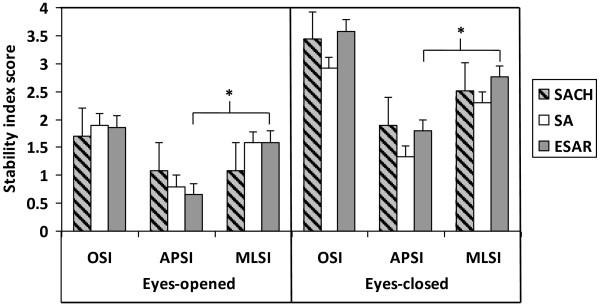
**Overall (OSI), anterior-posterior (APSI) and medial-lateral (MLSI) stability indexes score in mean (± standard error) between prosthetic foot types during eyes-opened and eyes-closed conditions.** The asterisk sign indicates statistically significant differences (p < 0.05) between APSI and MLSI within the same visual condition.

**Figure 3:**
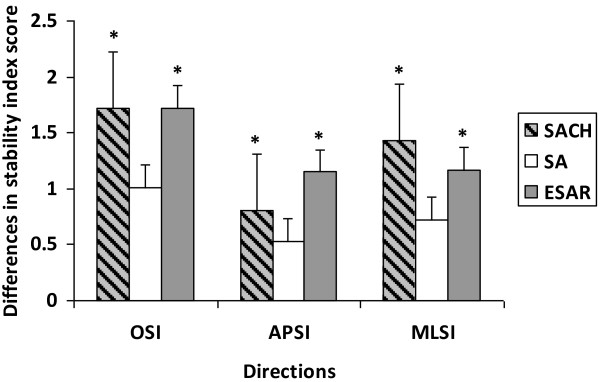
**Differences of overall (OSI), anterior-posterior (APSI) and medial-lateral (MLSI) stability index score between eyes-closed and eyes-opened conditions in mean (± standard error) according to prosthetic foot type.** The asterisk sign indicates statistically significant differences (p < 0.05).

Comparative Wilcoxon-signed rank analysis between visual conditions (EO-EC) revealed that the OSI, APSI and MLSI score were higher during eyes-closed compared to that of eyes-opened condition for all foot types (Figure 2). However the differences of stability scores between the two conditions were only statistical significant in OSI (p = 0.018) and MLSI (p = 0.018) for SACH foot, as well as in OSI (p = 0.043) and APSI (p = 0.027) for ESAR foot. Differences of stability scores for SA foot failed to reach any significant differences during eyes-closed and eyes-opened conditions (Figure 3).

### Perceived-balance assessment

The analysis of ABC score demonstrated a statistically significant differences between the SACH, SA and ESAR foot (p = 0.016). Further post-hoc analyses revealed that the differences occurred between ESAR and SACH (p = 0.043) as well as ESAR and SA (p = 0.028).

## Discussion

In this study, the influence of three prosthetic foot types to the postural steadiness in person with below-knee amputation was assessed during unperturbed standing. Additionally, the contribution of visual information in maintaining postural balance was evaluated. We demonstrated the possibilities of using Biodex stability system to provide clinical static balance assessment before progression into dynamic testing and training for populations with lower-limb amputation. Moreover, static balance has become an essential skill in rehabilitation process for the amputee populations to achieve independent standing and walking [1,9].

Prosthetic foot was prescribed to help amputees regulate the body’s CoM within the base of support to achieve postural equilibrium during quiet standing, as opposed to the plantarflexors-dorsiflexors mechanism in able-bodied person [5]. The primary findings in our study revealed that the control of postural steadiness during unperturbed bilateral standing was unaffected by the types of prosthetic foot used. Nevertheless, it is important to note that the SACH foot scored the lowest OSI indicating the least body sway when standing with the eyes-opened. This result may be due to the rigid ankle which offers no articulation thus minimizing the excursion of the CoM. Additionally, it further supports the notion from previous study that stiffer prosthetic foot maybe a potential justification in enhancing the safety of postural stability in this population by decreasing the body sway [11]. Similarly, our results were in accordance with previous study which proposed that the CoM excursion may have been constrained by the stiffness of the prosthetic ankle complex [12]. However, our study did not quantify the contribution from the intact limb or musculature of the residual limb which may influence the control of postural steadiness [9].

In contrast, the SA foot was considered most stable compared to other types of feet when visual input was removed as indicated by the lowest OSI. This finding suggested that the elimination of visual will increase utilization of other source of sensory information input in the organization of postural control. Particularly, the residual limb has been suggested to enhance the limited proprioceptive information [12] as the body weight is transmitted to the soft tissues via the socket to control the postural responses initiated at the ankle joint [5]. Additionally, the proprioception input from residual limb muscles may cause some movements at the ankle joint in the SA foot to counterbalance the body’s natural fluctuation in response to gravity during quiet standing. Our results agreed with the suggestion of prosthetic ankle range of motion as an important characteristic in foot-ankle component selection [24].

In able-bodied person, the lateral stability is controlled by alternating the activation of the hip abductors and adductors in order to transfer the body’s CoM between the legs [25]. However, lower limb amputation leads to insufficient control of weight-shifting to maintain posture which has caused instability in medial-lateral direction. We found that the deviation of CoM was greater in frontal plane as depicted by higher MLSI scores compared to APSI scores in both eyes-opened and eyes closed conditions for all foot types. The results of our study were in agreement with previous findings that an increase of CoM excursion in the medial-lateral direction maybe the results of compensation strategy to the impairment in controlling balance in the anterior-posterior direction [24]. However, the MLSI was significantly higher than APSI score in ESAR foot during eyes-opened and eyes-closed. This may be possibly due to the flexibility of the carbon fibre ESAR foot which provides eversion and inversion causing more sway movement and instability to the most of the subjects where single-axis foot is their habitual prosthesis. Additionally, the fear of falling which often occurs among the amputees can also lead to additional use of the hip strategy [26], which is reflected by the high stability indexes in medial-lateral direction in all prosthetic feet. Therefore, the medial-lateral instability experienced by the amputees can be utilized as a predictor for risk of falling [27]. This finding highlights the importance of learning how to balance over the prosthetic foot in order to control the displacement of CoM over the base of support for the amputees. Our results suggest that it is necessary to validate the improvement of postural stability in frontal plane following fall prevention program among the amputees.

Vision has been suggested as the main source of information used in the regulation of posture control under normal situation [4]. The findings of this current study corroborate with previous studies on amputees that showed greater postural instability when visual cues was occluded [28]. Explicitly, regardless of foot type, this study showed that the stability indexes were higher during eyes-closed condition which indicated greater deviation of CoM. The differences in balance indices between eyes-opened and eyes-closed conditions were only significance for SACH and ESAR, suggesting habitual adaptation to SA foot for most of the subjects.

Significant differences in the ABC scores found between prosthetic feet suggested that the amputees perceived disparities between the passive stability offered by the ankle mechanisms. Their perceived balance confidence was the highest in ESAR foot, followed by SA and SACH foot. This finding may be due to improved gait performance in lower-limb amputees such as increased tibial forward progression and adaptability to uneven terrain when using ESAR foot as reported previously [24,25].

We acknowledged that lack of previous studies comparing the influence of prosthetic foot types on the control of postural stability limits the possibility to associate our results with others. In addition, variations found in the length of residual limb among the subjects may affect postural stability where shorter residual limb exhibited larger body sway than that of medium length [29]. Additionally, the current results are only indicative for lower limb amputees whom are typical community ambulator and may not be generalized to all amputees. While the present study assessed balance control during quiet standing, future research should investigate the response of different prosthetic feet during more challenging situations to resemble real life situations. Results in this study were based on balance performance from a mixture of traumatic and diabetes caused of amputation. Researchers reported that person with amputation due to vascular adopted different balance control strategy with those of non-vascular reason due to poor somatosensory status found in dysvascular amputees, which caused an increase of body sway during quiet standing [30]. Therefore, larger sample size with similar characteristics might find a statistically significant difference in terms of postural control between prosthetic foot designs.

## Conclusion

The current study demonstrated that prosthetic foot types did not influence the maintenance of postural steadiness in below-knee amputees although there was a trend of better stability with rigid ankle foot. Nevertheless, visual cues were shown to affect postural stability in SACH and ESAR foot. This initial finding should be considered when prescribing the prosthetic foot to the amputees.

## Abbreviations

ABC: Activities-specific balance confidence; APSI: Anterior-posterior stability index; BBS: Berg balance score; BSS: Biodex stability system; CoM: Center of mass; EC: Eyes closed; EO: Eyes opened; ESAR: Energy storage and return; MLSI: Medial-lateral stability index; OSI: Overall stability index; SA: Single-axis foot; SACH: Solid ankle cushion heel foot; SOT: Sensory organization test.

## Competing interests

The authors declare that they have no competing interests.

## Authors’ contributions

NA designed the study concept, conducted the experiments, analyzed and interpreted the data as well as drafted the manuscript. NAAO and WABWA applied the financial grant, supervised the overall study and revised the manuscript draft. SA collected and analyzed the data and helped prepared the results and discussion of the manuscript. All authors read and approved the final manuscript.
